# Prediction Impairment May Explain Communication Difficulties in Autism

**DOI:** 10.3389/fpsyg.2021.734024

**Published:** 2021-09-28

**Authors:** Iris Scholten, Catharina A. Hartman, Petra Hendriks

**Affiliations:** ^1^Center for Language and Cognition Groningen, University of Groningen, Groningen, Netherlands; ^2^Department of Psychiatry, Interdisciplinary Center Psychopathology and Emotion Regulation, University of Groningen, University Medical Center Groningen, Groningen, Netherlands

**Keywords:** autism spectrum disorder, language processing, predictive processing, predictions, prediction impairment, language and communication difficulties

## Introduction

A central research question on autism is how the communication difficulties of autistic individuals can be explained. In this opinion paper we put forward the hypothesis that autistic individuals have problems with language because of an underlying impairment in the ability to generate and update predictions about language. Our hypothesis combines well-established findings from the past decade indicating that linguistic predictions facilitate faster language processing with recent evidence suggesting that autistic individuals show abnormalities in predictions outside the field of language. Investigating linguistic predictions in autism can help clarify the mechanisms underlying the communication difficulties of individuals with ASD. Our hypothesis subsumes earlier mechanistic explanations involving theory of mind and executive functions.

## Communication in Autism

Communication difficulties are a core component of autism. Research on communication typically distinguishes between structural language (i.e., the form and meaning of words and sentences) and pragmatic language (i.e., the use of language in social situations). In the past, many researchers assumed that autistic individuals mainly had problems with pragmatic language, such as irony (e.g., Happé, 1994; Leekam and Prior, 1994) and metaphors (e.g., Happé, 1994; Martin and McDonald, 2004). Some studies showed, however, that they have difficulties with structural language too (e.g., Brynskov et al., 2017; Wittke et al., 2017). For example, autistic individuals were found to have difficulties with which-questions (Prévost et al., 2017) and object relative clauses (Durrleman et al., 2015). Communication difficulties have been attributed to, among others, problems with theory of mind (ToM) or reduced executive functioning (EF), but these explanations do not speak to language problems beyond pragmatics. Additionally, our progress in gaining knowledge about ToM and EF in autism seems to have stagnated. We see this partly as a consequence of most studies using offline tasks, which only measure the ultimate response in a task but do not provide measures of the processes leading to this particular response (e.g., different cognitive processes can lead to the same outcome regarding ToM judgements or EF responses). Moreover, heterogeneity in outcomes (i.e., not every autistic individual shows inaccurate ToM judgements or EF responses; e.g., Baez et al., 2020; Deschrijver and Palmer, 2020) cannot be well-understood if the processes leading to these responses are not studied too. Thus, rethinking the theoretical foundations beyond ToM and EF is needed to accommodate structural language problems in autism. In addition, online measures (e.g., eye movements or brain activity) linked to the corresponding offline responses will foster insight into why some autistic individuals tend to have pragmatic and structural language problems, whereas others do not.

## Recent Developments

### Predictions in Language

Communication is fast and full of ambiguity. Thus, comprehenders must keep up with the speed of language and at the same time determine the intended meaning of a sentence (Crocker, 2005). Generating predictions are fundamental herein. Predictions about upcoming language speed up processing and thus help comprehenders to keep up with the speed of spoken language (e.g., Corps et al., 2018, 2019; Fitz and Chang, 2019; Kochari and Flecken, 2019; Shain et al., 2020).

It has been well-established that linguistic predictions are generated when particular linguistic information is activated in language users, even before the input that carries this information becomes available (Pickering and Gambi, 2018). For example, when hearing “John wants salt and pepper on his steak” a comprehender would be highly likely to predict lexical information, i.e., the word “pepper,” after hearing “salt and” before actually hearing “pepper,” because “salt and pepper” is a pair of words frequently used together in a fixed order. Preactivation of linguistic information is also empirically demonstrated by, for example, eye-tracking studies in which participants heard a sentence like “The boy eats the cake” while looking at pictures. Adults and children already look at the correct picture of a cake instead of competing pictures of other objects before they actually hear the word “cake” (e.g., Altmann and Kamide, 1999; Nation et al., 2003; Borovsky et al., 2012; Mani and Huettig, 2012). Prediction of the word “cake” comes from the verb “eats” which requires an object that is edible. This shows that comprehenders use lexical information to predict upcoming language.

Comprehenders also predict upcoming language on the basis of syntactic information. For example, Lukyanenko and Fisher (2016) showed that 3-year-old children are already faster and more likely to shift their gaze from an incorrect picture displaying only one thing to a correct picture displaying multiple things upon hearing a verb that requires a plural noun (e.g., “Where *are* the cookies?”) compared to a verb that is uninformative about the number of the noun (“Do you *see* the cookies?”). Thus, toddlers use agreement between the number marking (singular or plural) of a verb and a noun to predict features of an upcoming noun, resulting in a faster identification of the correct picture. The studies described above show that preactivation of lexical as well as syntactical information helps to do some of the processing ahead of time so that comprehenders can process language fast, despite the speed with which sentences are produced and despite the pervasive ambiguity of language (Pickering and Gambi, 2018).

Given the ambiguity in language and the need for fast processing, predictions can sometimes also steer comprehenders in the wrong direction. This is the case if a comprehender's initial prediction turns out to be false. For example, in a sentence like “The horse raced past the barn fell” (Bever, 1970), comprehenders will predict that “the horse” is the subject of the sentence, because there is a tendency to interpret the first noun phrase in a sentence as the subject, and subjects as agents. Subsequently, they will predict that “raced” is the sentence's main verb (referring to what the horse did) and that “past the barn” is the direction in which the horse raced. Thus, after hearing “the horse raced past the barn,” comprehenders will predict that the sentence is complete. However, upon hearing the final word of the sentence (the verb “fell”) comprehenders will discover that the sentence was not yet complete, and that “raced past the barn” was a specification of “the horse” (i.e., the horse that was raced past the barn). That is, comprehenders will discover that they were led up the garden path. These so-called garden path sentences thus require comprehenders to update their initial incorrect prediction by replacing it with a new prediction, to arrive at the intended interpretation of the sentence.

### Predictions Are Related to ToM and EF

Autistic individuals often have difficulties with language and communication. It is conceivable that these difficulties are a consequence of problems with predictions, and that the difficulties with ToM and EF often observed in autistic persons are linked to these prediction problems. ToM enables comprehenders to deduce why a person acted in a certain way or to anticipate how a person is likely to act. In this sense, ToM tasks are inherently prediction tasks, as comprehenders need to predict the actions of a person following a certain observation. Therefore, difficulties with ToM may be caused by a reduced ability to predict another person's behavior. EF is related to predictions as well, especially in situations where an initial prediction turns out to be incorrect. In such situations, comprehenders are required to inhibit their initial but false prediction and switch to an alternative interpretation while holding all information active in their working memory. Therefore, updating an incorrect predictions requires EF, in particular, cognitive inhibition (MacLeod, 2008), cognitive flexibility (Miyake and Friedman, 2012) and working memory (Miyake and Shah, 1999). This thus shows how ToM and EF are related to generating and updating predictions. However, as said earlier, the language difficulties seen in autism are broader than the hypotheses of ToM and EF can explain individually or together. In contrast, prediction impairments during language processing may explain the pragmatic and non-pragmatic language difficulties seen in autism and subsume the hypotheses of ToM and EF. Hence, ToM and EF are related to predictive abilities, but the process of generating and updating predictions is broader and has more explanatory power.

### Predictions Beyond Language

Recent studies have illustrated abnormalities in predictive abilities in autistic individuals outside the domains of language (see Cannon et al., 2021, for a recent review of empirical evidence). Neuroimaging and eye-tracking studies using tasks in which participants are presented with predictable repetitive stimuli that are infrequently interrupted by an unpredictable deviant stimulus found that autistic individuals showed an altered response compared to neurotypical controls (Jeste et al., 2015; Balsters et al., 2017; Lawson et al., 2017; Goris et al., 2018). The results of these studies suggest that autistic individuals may be less surprised when their predictions are being violated, as indicated by reduced brain responses (e.g., Lawson et al., 2017). This finding has been taken to mean that their predictions are less strong compared to those of neurotypical individuals (Pellicano and Burr, 2012), potentially because they struggle to generate precise internal prior beliefs about the world (both social and non-social; Pellicano and Burr, 2012; Friston et al., 2013; van de Cruys et al., 2014; Lawson et al., 2017), especially in temporally demanding environments where the environment changes fast in time (e.g., Pellicano and Burr, 2012; Hohwy et al., 2016; Vogel et al., 2019). Being less surprised after encountering a violation to a prediction could, in turn, mean that it is also harder to update a prediction, because the main function of surprise is to interrupt an ongoing action and reorient attention to the new, possibly significant stimulus (Kalat, 2015). This fits well with the finding in the language domain that predictions allow for faster processing. Indeed, the abnormalities in predictive abilities within autistic individuals found outside the language domain triggered our question if these abnormalities also occur within the language domain.

## Our Proposal

To explain the language and communication problems of autistic individuals, we put forward the Linguistic Prediction Impairment Hypothesis. This hypothesis states that autistic individuals show abnormalities in generating and updating predictions about language and can be seen as the linguistic version of a more general hypothesis about predictive processing differences between autistic individual and neurotypicals (see Pellicano and Burr, 2012; Sinha et al., 2014; van de Cruys et al., 2014). We hypothesize that this explains why autistic individuals:

- process language slower than their neurotypical peers (e.g., Kamio et al., 2007; Henderson et al., 2011; Bavin et al., 2014; Arunachalam and Luyster, 2018), as predictions facilitate faster language comprehension (Corps et al., 2018);
- have more problems with pragmatic language than structural language (e.g., Ludlow et al., 2017), as pragmatic language is more influenced by contextual information, making it less predictable;
- have difficulties with structural language nonetheless (e.g., Brynskov et al., 2017; Prévost et al., 2017; Wittke et al., 2017), as predictions are generated at every level of language (sounds, words, sentences and their meanings);
- have particular problems interpreting language when the initial prediction turns out to be incorrect (as is evidenced by the findings of Durrleman et al., 2015; Martins et al., 2018; Sukenik and Friedmann, 2018; Was et al., 2018), as updating predictions requires EF which is often found to be impaired in autistic individuals;
- show individual variability in their linguistic performance (e.g., Pearson and Hodgetts, 2020), as predictive abilities may vary strongly in autistic individuals;
- tend to have difficulties with ToM tasks as well as linguistic tasks requiring speaker-hearer coordination, as predictions about other people's actions are needed to succeed in these tasks (Schuwerk et al., 2016).

## Concluding Remarks

In this opinion paper, we have put forward the Linguistic Prediction Impairment Hypothesis, which states that autistic individuals have difficulties with language because they have difficulties with generating and updating predictions about language. This hypothesis could provide directions for further research and lead to new insights on language processing in autism, especially when focusing on the identification of subtle effects of predictions using methods that capture language processing online. While focused on the language and communication difficulties of autistic individuals, our proposal has broader implications. Prediction generation and prediction updating are less needed in restricted and repetitive situations. In such situations the future is more predictable and autistic individuals may therefore prefer such situations and behaviors (see also Pellicano and Burr, 2012; van de Cruys et al., 2014; Hohwy et al., 2016; Vogel et al., 2019). This allows for the integration of the DSM-5 diagnostic criteria of (A) impairments in social communication and interaction and (B) restricted, repetitive behaviors (American Psychiatric Association, 2013). Thus, our proposal would result in a more unified view of the core features of autism. Moreover, by emphasizing the importance of using online tasks measuring language processing instead of offline tasks merely registering the ultimate response, our proposal may lead to a better insight in the cognitive processes underlying linguistic behavior and may additionally increase our insights in the role of ToM and EF in generating and updating predictions about language.

## Author Contributions

IS wrote the first draft of the manuscript. CH and PH gave feedback on the first draft. All authors contributed to writing and revising the manuscript and read and approved the final version.

## Funding

This research was supported by grants from the Netherlands Organization for Scientific Research NWO (PhD in the Humanities Grant no. 322-89-012, IS).

## Conflict of Interest

The authors declare that the research was conducted in the absence of any commercial or financial relationships that could be construed as a potential conflict of interest.

## Publisher's Note

All claims expressed in this article are solely those of the authors and do not necessarily represent those of their affiliated organizations, or those of the publisher, the editors and the reviewers. Any product that may be evaluated in this article, or claim that may be made by its manufacturer, is not guaranteed or endorsed by the publisher.
